# The resolution of carotid arterial thrombus by oral anticoagulation after IV thrombolysis for chemotherapy-induced stroke: A case report

**DOI:** 10.1097/MD.0000000000021922

**Published:** 2020-08-28

**Authors:** Bo-Chang Wu, Ming-Hung Hu, Vinchi Wang

**Affiliations:** a School of Medicine, College of Medicine, Fu-Jen Catholic University, New Taipei City; b Division of Hematology and Oncology, Department of Medicine, Taipei Municipal Wangfang Hospital; c Taipei Cancer Center, Taipei Medical University, Taipei; d Department of Neurology, Cardinal Tien Hospital; e Medical Quality Management Center, Cardinal Tien Hospital, New Taipei City, Taiwan.

**Keywords:** arterial thromboembolism, dabigatran, thrombolysis, tissue plasminogen activator

## Abstract

**Rationale:**

Cancer-related stroke has been regarded as an emerging subtype of ischemic event. Acute treatment for this subtype may include the antiplatelet agents, anticoagulants, or endovascular intervention.

**Patient concerns:**

A 63-year-old woman with sudden-onset right hemiparesis and conscious change was sent to our emergency department. The patient had underlying sigmoid adenocarcinoma and received chemotherapy FOLFIRI (FOL, folinic acid; F, fluorouracil; and IRI, irinotecan) with targeted therapy cetuximab following lower anterior resection since the diagnosis was made.

**Diagnoses:**

Brain magnetic resonance angiography revealed a filling defect in left carotid bulb, and neurosonography showed a thick atherosclerotic plaque (size 4.9 mm) in the left internal carotid artery on day 5 after the onset of stroke.

**Interventions:**

During the first three hours after onset, administration of IV tissue plasminogen activator did not resolve the thrombus. Dabigatran (110 mg bid) started on day 7.

**Outcomes:**

The atherosclerotic plaque dissolved on day 24. The patient recovered her muscle strength but still had nonfluent speech in mild extent.

**Lessons:**

Thrombolytic and anticoagulant medications in this patient suggested the thrombus formation with fibrin-rich content which may be attributable to both cancer and chemotherapy. Dabigatran, an oral anticoagulant, had a benefit for this subtype of ischemic stroke among patients with cancer.

## 1 Introduction

Stroke may be a critical complication among patients with cancer, regardless of venous occlusion or arterial ischemia. It has been regarded as the most common cause of complex disability and the 3rd most common cause of death in Taiwan,^[1]^ and is still ranked the 2nd leading cause of death worldwide, despite the decline in the United States in the past decade.^[2]^ It is already known that early reperfusion therapy for acute ischemic stroke after administration by tissue plasminogen activator (tPA) has a better prognosis and is the mainstay of treatment for those without contraindications.

Cancer-related stroke has been regarded as an emerging subtype of ischemic event.^[3]^ Here, we report the case of a 63-year-old female patient with cancer with acute cerebral arterial embolism, in which the thrombus in the carotid bulb did not dissolve after tPA but did so later after dabigatran. There have been several case reports describing thrombus resolution after dabigatran, which acts as an anticoagulant by directly inhibiting thrombin.^[4–6]^ We will discuss some interesting issues regarding the pathogenesis and pharmacologic effects.

## 2 Case presentation

A 63-year-old woman presented to our emergency department with sudden-onset right hemiparesis and consciousness change. The patient got a diagnosis of sigmoid adenocarcinoma (pT3N1aM1, p-stage IV) 4 months before this stroke, and she had been on chemotherapy FOLFIRI (FOL, folinic acid; F, fluorouracil; and IRI, irinotecan) with targeting agent cetuximab as systemic therapy after lower anterior resection. Her last FOLFIRI chemotherapy (5th cycle) with cetuximab had been administered 4 days prior.

**Figure 1:**
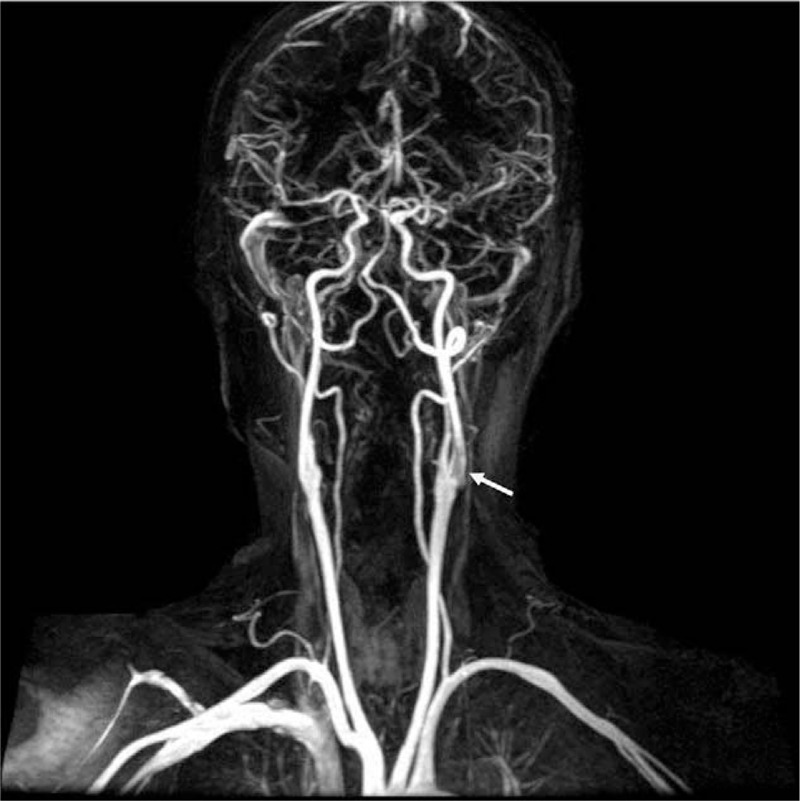
A filling defect (arrow) in left carotid bulb region by magnetic resonance angiography.

Sudden conscious change and fall took place 1 hour before arrival at our emergency room. Upon arrival, she was alert, normal vital signs, and the neurologic examination revealed right-sided limb weakness with speech problems. She exhibited nonfluent speech output, incorrect naming, word substitution, but intact repetition, fair comprehension, and obedience on secondary commands. The initial National Institute of Health Stroke Scale scored 6 out of 42. Brain computed tomography excluded intracranial hemorrhage, and then IV bolus of tPA (35 mg, 0.7 mg/kg) was administered soon. Mechanical thrombectomy was deferred because of her weak physical condition after the recent chemotherapy course. Echocardiography did not reveal any intracardiac thrombus. Brain magnetic resonance imaging showed acute infarction in left middle cerebral artery territory, mainly involving left insula and parietal lobe, and magnetic resonance angiography revealed a vague plaque image in left internal carotid artery (ICA) of the neck and occlusion of the Sylvian and cortical M3 segment of left middle cerebral artery (Fig. 1). Coagulation studies showed normal partial thrombin time, prothrombin time, fibrinogen, and antithrombin III, but elevated D-Dimer 1152 μg/mL. All other laboratory studies were within normal limits, including tumor marker CEA 1.19 ng/mL and CA-199 11.92 IU/mL. Her motor aphasia persisted, but her weakness improved during her stay in the ICU on the 2nd day.

**Figure 2:**
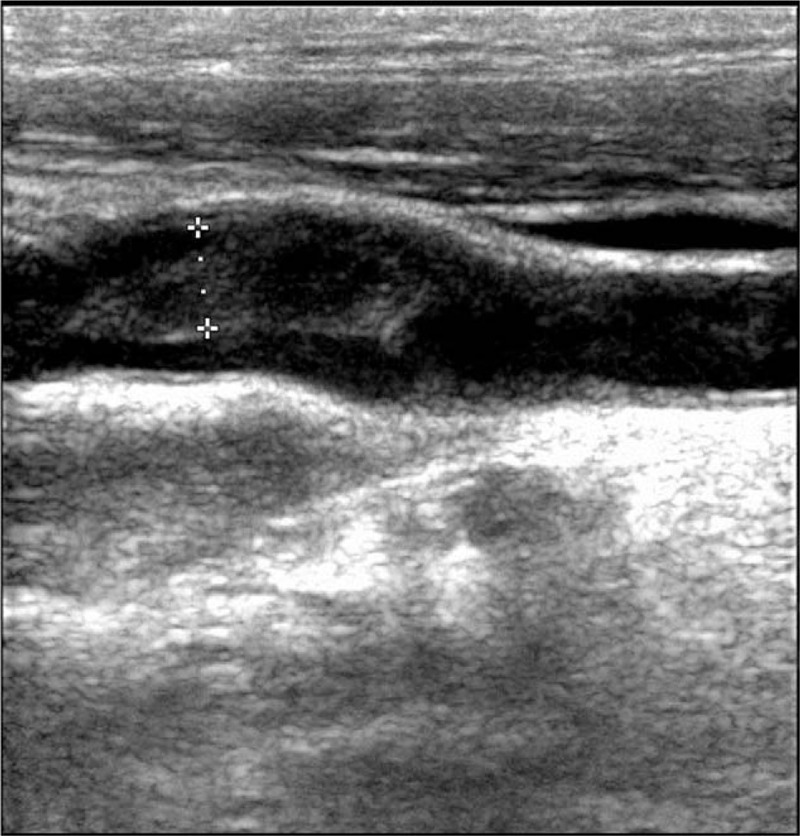
A thick thrombus (49 mm in size) in left carotid bulb on day 5 by neurosonography.

**Figure 3:**
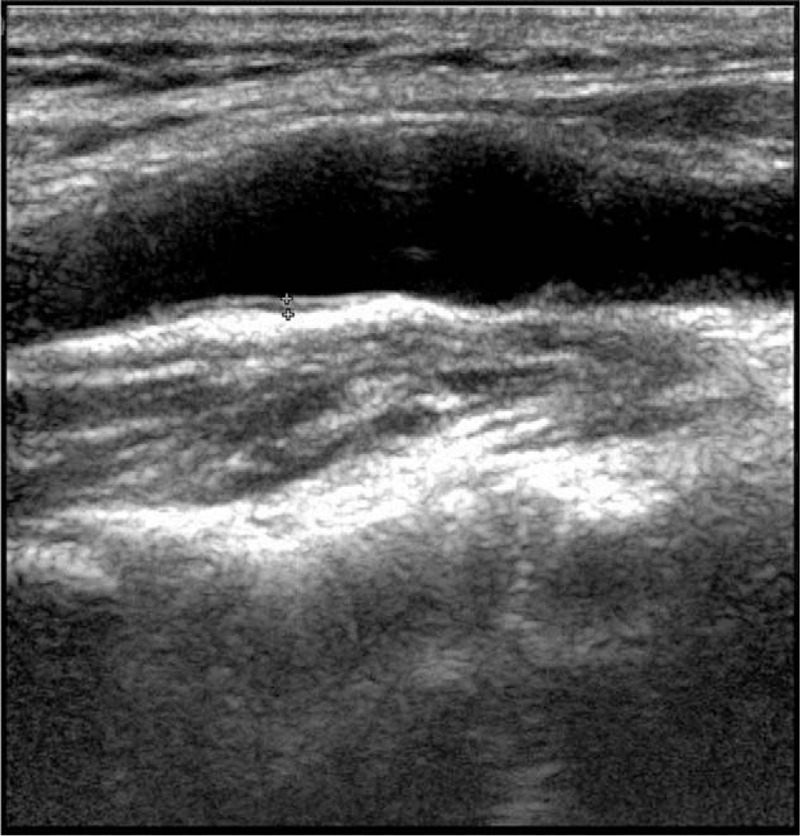
The thrombus dissolved on day 24 by the follow-up neurosonography.

On day 5 after the onset of stroke, neurosonography revealed a thick atherosclerotic plaque in left carotid bulb (Fig. 2). On day 7, she started dabigatran (110 mg twice daily) due to suspicion of cerebral embolism possibly caused by ICA plaque rupture. The plaque image by the follow-up neurosonography still existed on day 10, but disappeared on day 24 (Fig. 3), that is, 17 days after the use of dabigatran. During these 17 days, dabigatran was the only treatment administered. The patient left the hospital with mild motor dysphasia but no motor weakness. After discharge, the patient was on dabigatran for 3 months, and then aspirin to prevent thrombus formation. Besides, she continued oral chemotherapy Tegafur (5-FU) and remained stable for the following 18 months. There was no new episode of stroke during the follow-up period.

## 3 Discussion

We cast interest on this case because of the finding of atheroma by magnetic resonance angiography and neurosonography, and of the atheroma resolving after the use of dabigatran. Someone may question clinical issues about this case, including why the intravenous thrombolysis by tPA failed but later atheroma resolved by dabigatran; why the atheroma formed in the artery rather than vein; and whether the atheroma formation caused by cancer itself or by chemotherapy.

The tPA, a serine protease found on the endothelial lining of blood vessels, binds to fibrin and facilitates plasminogen conversion to plasmin, which then breaks down the thrombus.^[7]^ The efficacy of recanalization by tPA may be related to the location and burden of the thrombus, as the thrombus in the proximal ICA is more resistant to tPA than distal ones.^[8]^ In our case, we also postulate that tPA did have some subtle effect on the thrombus that made the latter medication – dabigatran resolve this atheroma efficiently. There have been several reports of similar resolution of intravascular and intracardiac thrombi by dabigatran. Kaku reported a case of intracardiac thrombus resolution after anticoagulation therapy with dabigatran (150 mg twice daily).^[4]^ Nagamoto et al reported an example that dabigatran (110 mg twice daily) resolved a left ventricular thrombus.^[5]^ Akiyama et al described the thrombolytic action of dabigatran (110 mg twice daily) on an ICA thrombus.^[6]^ Kaku and Nagamoto et al reported cases with the resolution of intracardiac thrombi, whereas Akiyama et al reported a carotid thrombus. The intracardiac thrombus dissolved completely in 21 and 27 days in Kaku's and Nagamoto's cases, respectively (Table 1). The patient in the report by Akiyama et al took irregular dosing of dabigatran 110 mg bid for approximately 3 years. A carotid thrombus was incidentally found by the neck vessel sonographic examination performed for cancer staging, without mentioning the thrombus size. Regular use of dabigatran began in that case, and the carotid thrombus disappeared in 16 days. There are some similarities between our and Akiyama's cases, including the thrombus location (both were within ICA near carotid bulb), the dose of dabigatran (both were 110 mg twice daily), and history of cancer (stage III esophageal carcinoma in Akiyama's patient and stage IV colorectal carcinoma in ours). All of the above case reports did not mention the use of tPA for their patients. Dabigatran was the only treatment for the thrombus.

**Table 1:**
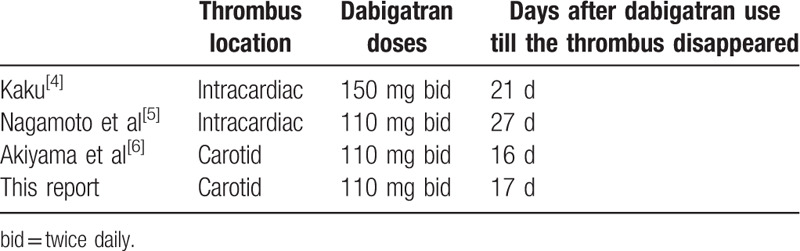
Thrombus resolution after dabigatran use, listing the thrombus location, dabigatran doses, and total treatment days of dabigatran before the thrombus disappearance first noted in our and previous reports.

Dabigatran, a direct oral anticoagulant, directly binds to thrombin. Without the activation of thrombin, the drug blocks the transformation of fibrinogen into fibrin, resulting in no new thrombus formation.^[9]^ Besides its primary action of anticoagulation, its contribution to thrombolysis is postulated to be the inhibition of thrombus extension, which in turn causes unstable fibrin networks to be broken down by plasmin.^[10]^

Thrombi can be classified as white or red thrombi based on their macroscopic appearance and predominant color. Several different descriptions of red and white thrombi have been published. Koupenova et al described white thrombus as a platelet-rich thrombus formed in arteries, and red thrombus as a fibrin- and erythrocyte-rich thrombus formed in veins due to different velocities in arteries and veins.^[11]^ In contrast, there are few reports of fibrin-rich thrombosis in arteries.^[12,13]^ According to Quadros et al, white thrombi have a higher percentage of fibrin but a lower percentage of erythrocytes than red thrombi.^[14]^ White thrombi are smaller and mainly composed of fibrin. Maekawa et al reported that cardioembolic thrombi have a higher percentage of fibrin and fewer erythrocytes than noncardioembolic thrombi.^[15]^ Therefore, white thrombi may be either platelet rich or fibrin rich, whereas red thrombi contain erythrocytes.

We did not obtain the thrombus sample for pathologic diagnosis from this patient. Nevertheless, based on the fact that the thrombus disappeared after dabigatran administration, we thought that a fibrin- and erythrocyte-rich red thrombus was more likely than a platelet-rich white one in our case. According to Maekawa et al, a higher percentage of erythrocytes was more common in a noncardioembolic thrombus,^[15]^ which was consistent with the left ICA thrombus in our case. Dabigatran would resolve the plaque indirectly by limiting the extension of the existing thrombus, making it easier to be removed by plasmin.

Another interesting issue is whether chemotherapy or cancer itself caused thrombus formation. Navi et al compared the incidences of arterial thromboembolisms (ATEs) (such as stroke and myocardial infarction) in patients with cancer at different sites and among those with advanced stages.^[16]^ It is known that the risk of ATEs is higher in lung, colorectal, and gastric cancers. Advanced stages further increase the risk, and the risk of ischemic stroke is highest within the first 6 months after the diagnosis of cancer. The cumulative incidences of myocardial infarction and ischemic stroke since the diagnosis of colorectal cancer have been reported. Six-month hazard ratios of myocardial and cerebral ischemia among newly diagnosed patients with cancer were estimated to be 2.9 and 1.9, respectively.^[16]^ It seems that intensive chemotherapy after diagnosis might be associated with this increased risk, as in our case.

The multifactorial pathogenesis of ATEs in cancers may include individual cardiovascular risks, comorbidities, immobilization, primary cancer location, and treatment by chemotherapy or radiotherapy.^[17]^ Cancer can activate platelets, increase expression of procoagulants, and suppress fibrinolytic activity.^[18–20]^ Activation of these procoagulant factors can take place during the apoptosis of tumor cells induced by chemotherapy or radiation.^[17]^ Both chemotherapy and radiotherapy cause endothelium damage, which induces the activation of platelets and the coagulation cascade. In addition, a reduction in fibrinolysis is involved in tumor growth and metastasis. Furthermore, 5-fluorouracil, a pyrimidine analogue used in the treatment of colorectal cancer, may cause direct endothelial damage leading to platelet and coagulation cascade activation.^[17]^

In this patient with colorectal cancer, it was unlikely that the stroke was the result of metastasis or arterial invasion of tumor growth based on the evidence that dabigatran resolved the thrombus. Both cancer itself and chemotherapy give rise to a hypercoagulable state; therefore, it is difficult to say which one induced the thrombus. However, we figured that chemotherapy was the primary factor in thrombus formation because the onset of stroke was within a short period after the chemotherapy courses. We did not perform IA thrombectomy for this patient. If we did, the content of thrombus by pathologic analysis, platelet- or fibrin-rich, would cast more interest in the pathogenesis of the thrombus.

Thus, in our case, we hypothesized a “two-step” mechanism in this cancer-related stroke. First, the cancer made an environment with higher procoagulants predisposing the patient to thrombus formation. Second, on the following adjuvant chemotherapy, accelerated thrombus formation by damaging the endothelium and inducing the coagulation cascade occurred. Given that our patient developed stroke after several cycles of chemotherapy, the diagnosis of chemotherapy-induced stroke was reasonable as these events occurred chronologically. We also observed the thrombus resolution effect of dabigatran, which could be explained as stated in this article. In this stroke patient with colon cancer, thrombus formed in a “two-step” mechanism, which was later resolved by an oral anticoagulant. Oral anticoagulation had benefit for this subtype of ischemic stroke. Thrombolytic and anticoagulant medications in this patient suggested the thrombus formation with fibrin-rich content which may be attributable to both cancer and chemotherapy.

## Acknowledgment

The authors thank Editage-Taiwan (#VIANG_4) for providing this editing service.

## Author contributions

Bo-Chang Wu: review of the medical history; literature search; manuscript drafting.

Ming-Hung Hu: cancer history review; consultation of medication effects.

Vinchi Wang: stroke patient care; draft review and discussion; literature search.
